# Heat Shock Factor HSFA6b Mediates Mitochondrial Unfolded Protein Response in *Arabidopsis thaliana*

**DOI:** 10.3390/plants13223116

**Published:** 2024-11-05

**Authors:** Guolong Yu, Zhuoran Huang, Chaocheng Guo, Jiahao Li, Xinyuan Wang, Yudong Wang, Xu Wang

**Affiliations:** Shanghai Collaborative Innovation Center of Agri-Seeds, Joint Center for Single Cell Biology, School of Agriculture and Biology, Shanghai Jiao Tong University, Shanghai 200240, China

**Keywords:** mitochondria, mitochondrial proteotoxic stress, mitochondrial unfolded protein response UPR^mt^, heat shock factor, heat shock protein

## Abstract

Mitochondria are important organelles in eukaryotes and are involved in various metabolic processes. Mitochondrial proteotoxic stress triggers the mitochondrial unfolded protein response (UPR^mt^) to restore mitochondrial protein homeostasis and maintain normal life activities. However, the regulatory mechanism of plant UPR^mt^ remains to be revealed in Arabidopsis. Based on the fact that UPR^mt^ activates heat shock protein (*HSP*) genes, we identified the heat shock transcription factor *HSFA6b* as a key regulator mediating UPR^mt^ through reverse genetics. *HSFA6b* responded to mitochondrial proteotoxic stress and regulated mitochondrial heat shock proteins’ genes’ (*mtHSPs*) expression. HSFA6b translocated to the nuclear after treatment with doxycycline (Dox)—a mitochondrial ribosome translation inhibitor. HSFA6b binds to the *mtHSPs* promoters and activates *mtHSPs* expression. The *HSFA6b* mutation blocked the UPR^mt^ signals to promote root growth under mitochondrial proteotoxic stress and accelerated leaf senescence during development. Our study reveals a novel signal-regulating mechanism in the UPR^mt^ pathways and provides new insights regarding the regulation of plant growth and development and stress resistance by the UPR^mt^ pathways.

## 1. Introduction

Mitochondria are important organelles in eukaryotes and are involved in various metabolic processes. As the energy factory of cells, mitochondria produce a large amount of ATP as the main energy source for all life activities [1]. During the process of energy conversion, mitochondrial dysfunction, caused by drastic changes in the external environment, destroys the folding and assembly of mitochondrial proteins and induces mitochondrial proteotoxic stress [2]. Under mitochondrial protein toxicity stress, mitochondria communicate with the nucleus through specific signaling molecules. This triggers an increase in the transcription of nuclear-encoded mitochondrial chaperones and proteases, which help maintain mitochondrial protein homeostasis. This protective stress response within the cell is known as the mitochondrial unfolded protein response (UPR^mt^) [3,4,5,6]. UPR^mt^ is a key component of mitochondrial retrograde signaling, activated by various types of mitochondrial dysfunction, including imbalances in the protein quality control system, accumulation of unfolded proteins, and disruptions in the electron transport chain. Its primary function is to reduce the accumulation of unfolded proteins in mitochondria and ensure mitochondrial protein homeostasis [7,8,9]. In addition, UPR^mt^ is involved in monitoring mitochondrial dysfunction and regulating the chromatin structure and gene transcription [10]. Research on the UPR^mt^ mechanism has mainly focused on yeast, nematodes, and mammalian cells, with relatively few studies exploring the mechanism in plants [9]. In our previous research, we demonstrated the existence of a conserved UPR^mt^ in plants and found that they regulate molecular chaperones, such as heat shock proteins (HSPs), mainly through reactive oxygen species and hormone signals to maintain mitochondrial protein homeostasis, which has a significant impact on plant growth and development [5]. However, the specific molecular regulatory mechanisms need to be further studied.

In C. elegans, UPR^mt^ is regulated by the transcription factors *ATFS-1*, *DVE-1*, *UBL-5*, the protease *ClpP*, and the ABC transporter *HAF-1* in different ways. Under normal conditions, ATFS-1 is located in mitochondria and degraded by mitochondrial protease Lon. When mitochondrial protein stress occurs, the abundance of ATFS-1 in the cytoplasm increases and then transfers to the nucleus to activate the expression of mitochondrial molecular chaperones, proteases, and other mtPQC-related genes to restore mitochondrial function [11]. In mammals, UPR^mt^ restores mitochondrial protein homeostasis by the transcription factors *CHOP* and *ATF5*, which regulate downstream mtPQC-related gene expression. *ATF5* showed a similar regulatory mechanism to *ATFS-1* in regulating UPR^mt^ activity [12]. In addition to maintaining mitochondrial protein homeostasis, UPR^mt^ is also involved in the monitoring of mitochondrial dysfunction, signal communication between mitochondria and the nucleus, and regulation of the chromatin structure and gene transcription [13]. At the ontogenetic level, UPR^mt^ is involved in the regulation of the life cycle and is closely related to the aging process of organisms [14].

Compared with the key regulatory genes of the UPR^mt^ signaling pathway, such as *ATFS-1*, *DVE-1*, and *UBL-5*, which have been found in yeast and animals [8,15,16], no *ATFS-1* homologous genes were found in the plant genome, suggesting that there may be a relatively specific signaling pathway for UPR^mt^. Recent studies indicate that the mitochondrial retrograde signaling transcription factor *ANAC017* plays a key role in regulating the UPR^mt^ signaling pathway [17]. Additionally, UPR^mt^ signaling activates the ABA pathway, which, through ABA Insensitive 5 (*ABI5*), enhances the expression of UPR^mt^ genes. More and more UPR^mt^ signaling pathways are gradually being uncovered [18].

Heat stress-induced and constitutively expressed conserved members of the *HSP* family serve as molecular chaperones, playing an essential role in maintaining and restoring protein homeostasis. Under stress conditions, a reduction in molecular chaperones leads to protein denaturation and impairs the processing of newly synthesized proteins. This cytoplasmic protein stress response activates the transcription of heat shock protein (*HSP*) genes through the action of heat shock transcription factors (*HSFs*), which are crucial for preserving cellular protein homeostasis [19,20]. During the heat shock response, HSFs primarily regulate the expression of *HSPs* by binding to heat shock elements (HSEs; nGAAnnTTCn), thereby activating *HSP* gene transcription [21]. In contrast to animals, plants have a greater number of *HSFs*, which function as activators of *HSPs*—except for *HSFB1* and *HSFB2b*—indicating that plants possess a more complex system for maintaining protein homeostasis [22,23]. During the activation of the UPR^mt^ signaling pathway in plants, mitochondrial-related *HSP* genes are significantly upregulated, yet the upstream *HSF* transcription factors responsible for this activation require further investigation and validation [5].

In this study, we identified *HSFA6b* as a novel signaling molecule in the mitochondrial UPR^mt^ pathway. Under mitochondrial proteotoxic stress conditions, HSFA6b translocates to the nucleus and activates the expression of the *mtHSPs* gene by binding to their promoter region; the *HSFA6b* mutation blocked the UPR^mt^ signals to promote root growth and accelerated leaf senescence during development.

## 2. Results

### 2.1. Screening of Transcription Factors in the UPR^mt^ Signaling Pathway

In previous studies of the UPR^mt^ signaling mechanism, it was found that in Arabidopsis with mitochondrial ribosomal protein mutations or chemical interference, the UPR^mt^ signaling pathway is activated, thereby promoting the expression of downstream *mtHSPs* genes [5]. We selected five *HSP* genes, *HSP60-2*, *HSP60-3A*, *HSP60-3B*, *mtHSC70-1*, and *mtHSC70-5*, as potential candidate reporter genes. Pre-experiments were conducted using the tobacco transient transformation system to test promoter activity. After treatment with doxycycline for 12 h, compared to the other four promoters, the promoter activity of *mtHSC70-5* was significantly enhanced. Therefore, *mtHSC70-5* can serve as a reporter gene for the mitochondrial UPR^mt^ signaling pathway (Figure S1).

**Figure 1:**
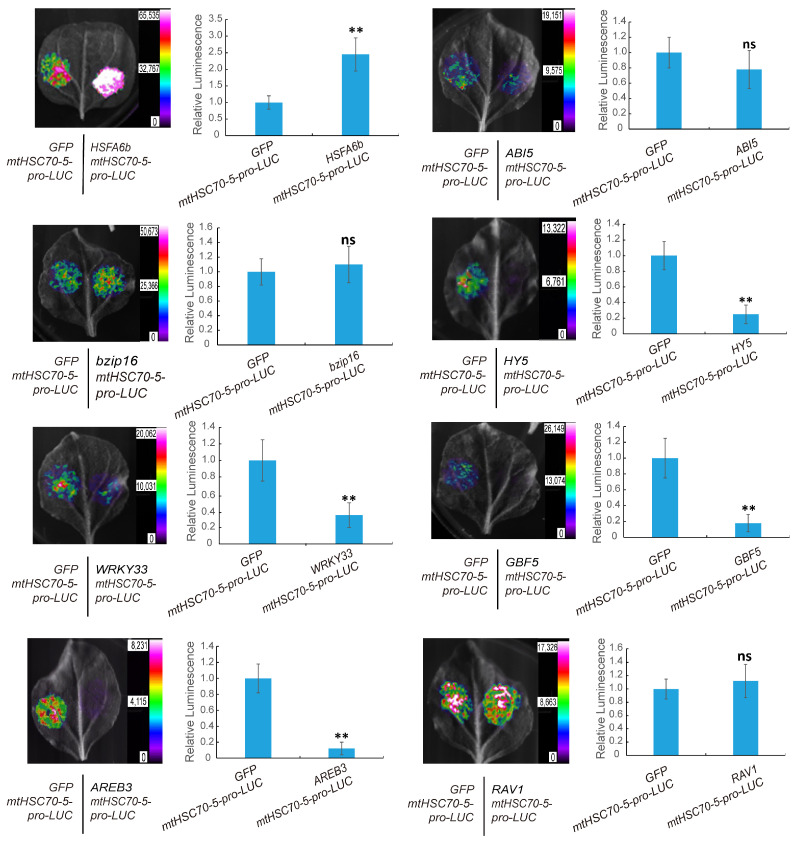
Screening of transcription factors that mediate retrograde signaling in UPR^mt^: In each transcription activation experiment, 10 tobacco leaves were injected. The images in the figure represent the experimental setup, with the mean relative fluorescence values plotted for the experimental and control groups. The experiment was conducted with three biological replicates, and paired two-sample *t*-tests on the average values were used to analyze significant differences (ns, *p* > 0.05; **, *p* < 0.01). Error values are presented as standard errors.

The transcription factors that may bind to the *mtHSC70-5* promoter region were analyzed using the Arabidopsis DAP-seq database in the Plant Cistrome Database [24]. Combining this analysis with previously published Arabidopsis transcriptome data after activation of the UPR^mt^ signal [5], a total of eight potential transcription factors were identified as candidates that mediated the activation of *mtHSC70-5* expression (Figure S2). Experimental validation of transcriptional activation was conducted, revealing that only *HSFA6b* exhibited strong activating effects on the *mtHSC70-5* promoter activity (Figure 1), while others showed inhibitory effects or no significant changes. Consequently, *HSFA6b* was selected as the transcription factor mediating the plant UPR^mt^ signal transmission for further functional validation.

### 2.2. The HSFA6b Mediates the Signal Transduction of Plant UPR^mt^

**Figure 2:**
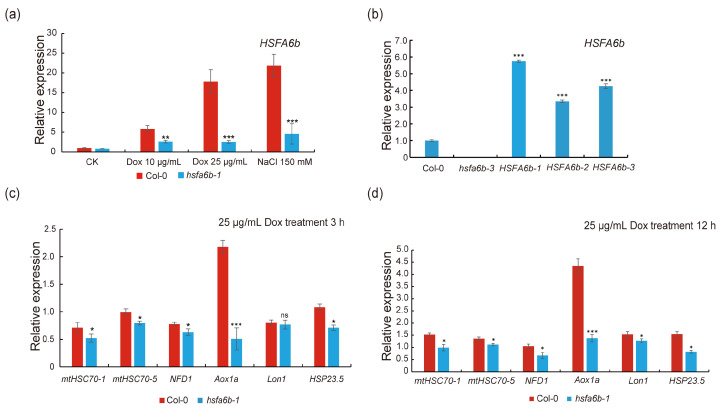
*HSFA6b* mediates the signal transduction of plant UPR^mt^: (**a**) *HSFA6b* responds to mitochondrial protein toxicity stress: Assessment of *HSFA6b* gene expression in Col-0 and *hsfa6b-1* mutants after seven days of germination treated with Dox and NaCl. NaCl was used as a positive control, as previous reports have shown that *HSFA6b* responds to NaCl treatment [21]. (**b**) The identification results of *HSFA6b* overexpression and *hsfa6b-3* mutant materials. (**c**) Expression of mitochondrial unfolded protein response-related genes in Arabidopsis Col-0 and *hsf6b-1* mutant seedlings after 3 h of treatment with 25 μg/mL of Dox. (**d**) Expression of mitochondrial unfolded protein response-related genes in Arabidopsis Col-0 and *hsfa6b-1* mutant seedlings after 12 h of treatment with 25 μg/mL of Dox. The Arabidopsis gene *UBQ10* serves as an internal reference gene, and the experiment was conducted with three biological replicates. Paired two-sample *t*-tests on the average values were used to analyze significant differences (ns, *p* > 0.05, * represents 0.01 < *p* < 0.05, ** represents 0.001 < *p* < 0.01, and *** represents *p* < 0.001); error bars indicate standard errors.

To evaluate the impact of the *HSFA6b* gene on the mitochondrial Unfolded Protein Response (UPR^mt^) signaling pathway. We measured the expression level of the *HSFA6b* gene 12 h after Dox treatment. Following 25 μg/mL of Dox treatment, the expression of the *HSFA6b* gene increased approximately 15–20-fold in the wild type (Col-0), but only about 3-fold in the *hsfa6b-1* mutant, indicating a strong transcriptional response of *HSFA6b* to mitochondrial proteotoxic stress (Figure 2a). Subsequently, we constructed the *HSFA6b* overexpression plants and detected their expression level by qRT-PCR. Compared with the wild type, the relative expression of the *HSFA6b* gene in the overexpression lines increased by 3–6-fold (Figure 2b). Furthermore, we scrutinized the expression profiles of the mitochondria-associated genes implicated in the unfolded protein response in Arabidopsis seedlings following doxycycline treatment. After 3 and 12 h of Dox treatment, the expression levels of the genes associated with the mitochondrial unfolded protein response, such as *mtHSC70-1*, *mtHSC70-5*, *HSP23.5*, *NFD1*, *Lon1*, and *Aox1a*, showed differing degrees of decrease in the treated *hsfa6b-1* mutant compared to the wild type (Col-0). Notably, the reduction in expression of the mitochondrial stress marker gene *Aox1a* was particularly pronounced (Figure 2c,d). Cumulatively, these research findings indicate that *HSFA6b* serves as a crucial mediator in the signal transduction of UPR^mt^, modulating the expression of specific genes within the UPR^mt^ signaling pathway.

### 2.3. HSFA6b Transfers into the Nucleus in Response to Mitochondrial Proteotoxic Stress

**Figure 3:**
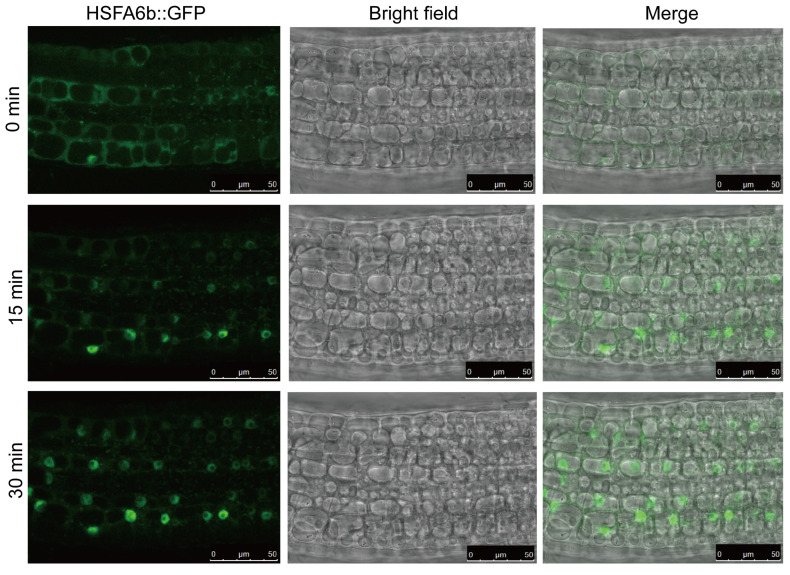
HSFA6b transfers into the nucleus to respond to mitochondrial proteotoxic stress: Confocal microscopy of *HSFA6b:GFP* Arabidopsis, which HSFA6b fused to the C-terminal GFP reporter gene. *HSFA6b:GFP* seeds were treated with ½ MS liquid medium containing 25 μg/mL of Dox. Root cells were observed using a Leica SP8 confocal laser scanning microscope with 488 nm excitation.

In previous studies, it was noted that HSFA6b translocates into the nucleus in response to ABA treatment, demonstrating nucleocytoplasmic shuttling characteristics [21]. To investigate the subcellular localization of HSFA6b under mitochondrial protein toxicity stress, five-day-old seedlings of *HSFA6b::GFP* overexpressing transgenic Arabidopsis were treated with 25 μg/mL of Dox dissolved in liquid ½ MS medium. Subsequently, subcellular localization was observed. After 15 and 30 min of Dox treatment, the fluorescence gradually concentrated in the nucleus (Figure 3), suggesting that under mitochondrial protein toxicity stress conditions, HSFA6b also exhibits nucleocytoplasmic shuttling characteristics to orchestrate the expression of downstream genes associated with mitochondrial protein homeostasis.

### 2.4. HSFA6b Binds to the mtHSPs Promoter and Activates mtHSPs Expression

**Figure 4:**
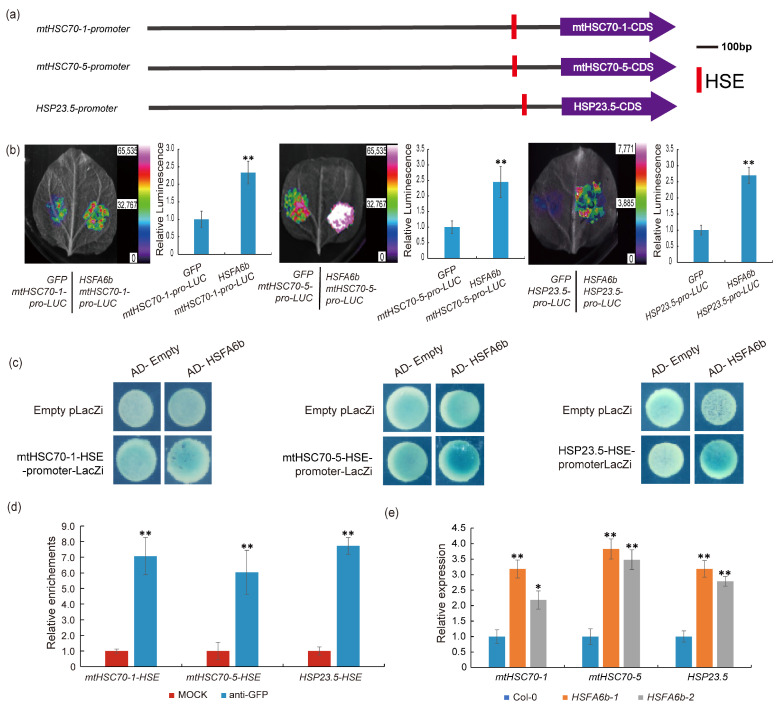
HSFA6b binds to and activates the promoter of *mtHSPs*: (**a**) Schematic diagram of the promoter region of *mtHSPs* (*mtHSC70-1*, *mtHSC70-5*, and *HSP23.5*). The red bar chart shows heat shock elements HSE (HSEs; nGAAnnTTCn) [21]. (**b**) The transcriptional activation experiment established the regulatory relationship between HSFA6b and *mtHSPs*. In each transcription activation experiment, 10 tobacco leaves were injected. The images in the figure represent the experimental setup, with the mean relative fluorescence values plotted for the experimental and control groups. (**c**) Yeast one-hybrid (Y1H) assay showing the interaction between HSFA6b and *mtHSPs*: yeast cells contain pB42AD, pB42AD-*HSFA6b*, and the HSE motifs region from the *mtHSPs* (*mtHSC70-1*, *mtHSC70-5*, and *HSP23.5*) promoter were grown on SD/-Trp/-Ura selective medium containing 20 mg/L X-gal. The Y1H assays were repeated three times, and representative images are shown. (**d**) Results of the ChIP-qRT-PCR analysis of the binding of HSFA6b to the *mtHSPs* (*mtHSC70-1*, *mtHSC70-5*, and *HSP23.5*) promoter, with the calculated relative enrichment used as the IP/Input. (**e**) Results of the qRT-PCR analysis of *mtHSPs* (*mtHSC70-1*, *mtHSC70-5*, and *HSP23.5*) transcript levels in the *HSFA6b*-overexpressing transgenic plants. The Arabidopsis gene, *UBQ10,* serves as an internal reference gene. The experiment was conducted with three biological replicates. Paired two-sample *t*-tests on the average values were used to analyze significant differences (* represents 0.01 < *p* < 0.05, ** represents 0.001 < *p* < 0.01); error bars indicate standard errors.

Through a transcriptional activation assay, the heat shock transcription factor *HSFA6b* was screened for its ability to activate *mtHSC70-5* expression, mediating the mitochondrial unfolded protein response (Figure 1 and Figure 2). To further validate the regulatory role of *HSFA6b* on the genes related to the UPR^mt^, we selected three *HSP* genes (*mtHSC70-1*, *mtHSC70-5*, and *HSP23.5*) that are more sensitive to mitochondrial stress for validation. We found conserved HSE (heat shock elements) in the promoter regions of these three *HSP* genes (Figure 4a). We used a tobacco transient expression assay to verify their regulatory relationship, and the results showed that *HSFA6b* significantly activated the promoter activity of these three mitochondrial *HSP* genes (Figure 4b). Subsequent yeast one-hybrid (Y1H) assays showed that HSFA6b can bind to the promoter regions of three mitochondrial *HSP* genes and activate the expression of the reporter gene (Figure 4c). We further used ChIP (Chromatin Immunoprecipitation) coupled with qRT-PCR analysis to verify the regulatory relationship between HSFA6b and *mtHSPs*, where the results showed that chromatin immunoprecipitated with anti-GFP antibody was profoundly enriched in the HSE regions of the *mtHSPs* promoters (Figure 4d). Additionally, the qRT-PCR results showed that in *HSFA6b*-overexpression plants, the expression levels of *mtHSC70-1*, *mtHSC70-5*, and *HSP23.5* were significantly upregulated compared to the wild type (Figure 4e). In summary, HSFA6b binds to the *mtHSPs* promoter and activates *mtHSPs* expression.

### 2.5. HSFA6b Mutation Promotes Root Growth Under Mitochondrial Proteotoxic Stress

**Figure 5:**
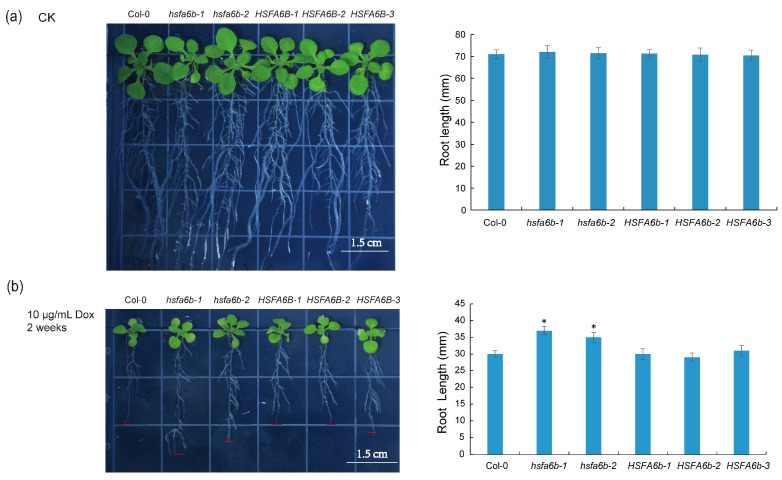
The root length phenotype of the *hsfa6b* mutants under mitochondrial proteotoxic stress: (**a**) The root length phenotype of Col-0, mutants (*hsfa6b-1, hsfa6b-2*), and overexpression plants (*HSFA6b-1, HSFA6b-2,* and *HSFA6b-3*) without mitochondrial proteotoxic stress (*n* = 12 plants). (**b**) The root length phenotype of Col-0, mutants (*hsfa6b-1*, *hsfa6b-2*), and overexpression plants (*HSFA6b-1*, *HSFA6b-2,* and *HSFA6b-3*) with mitochondrial proteotoxic stress (*n* = 12 plants). The red line in the left figure is used to mark the position of the root tip of the primary root. Asterisks indicate significant differences between Col-0 and mutants or overexpression lines (Student’s *t*-test; * 0.01 < *p* < 0.05).

In previous studies, the root length was inhibited after activating UPR^mt^ signaling (through the Dox treatment or *MPL1* gene mutation) [5]. To investigate the role of *HSFA6b* in the UPR^mt^ signaling pathway, we utilized *hsfa6b* mutants and overexpressing lines to observe the root length under the Dox treatment. We exposed 7-day-old Arabidopsis seedlings to 10 μg/mL of Dox in the ½ MS medium and recorded the root growth after 2 weeks. After 2 weeks of treatment, there were no differences observed among the wild type, mutants, and overexpression lines in the control group without Dox, while in the experimental group treated with Dox, the root length of the *hsfa6b* mutants increased compared to the wild type, the root length of the overexpression lines showed no significant change (Figure 5). Therefore, *HSFA6b* mutation promotes root growth under mitochondrial proteotoxic stress.

### 2.6. HSFA6b Mutation Induces Early Flowering and Premature Senescence in Arabidopsis

**Figure 6:**
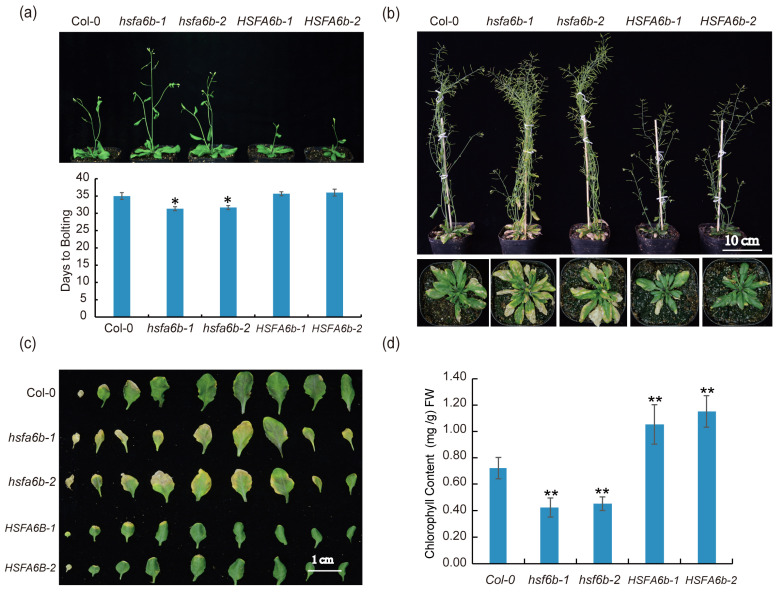
*HSFA6b* mutation induces early flowering and premature senescence: (**a**) The flowering phenotype of Col-0, mutants (*hsfa6b-1* and *hsfa6b-2*), and overexpression plants (*HSFA6b-1* and *HSFA6b-2*) (*n* = 12 plants), where the upper part of the image displays the flowering phenotype, while the lower part presents the flowering time data statistics; (**b**) the plant senescence phenotype of Col-0, mutants (*hsfa6b-1*, *hsfa6b-2*), and overexpression plants (*HSFA6b-1* and *HSFA6b-2*); (**c**) the leaf senescence phenotype of Col-0, mutants (*hsfa6b-1* and *hsfa6b-2*), and overexpression plants (*HSFA6b-1* and *HSFA6b-2*); (**d**) chlorophyll content of senescent leaves in Col-0, mutants (*hsfa6b-1* and *hsfa6b-2*), and overexpression plants (*HSFA6b-1* and *HSFA6b-2*) (*n* = 12 plants). Asterisks in (**a**,**d**) indicate significant differences between Col-0 and mutants or overexpression lines (Student’s *t*-test; * 0.01< *p* < 0.05, ** 0.001< *p* < 0.01).

The activation of the UPR^mt^ signaling pathway has been reported in previous studies to regulate the lifespan and delay aging [5,13,25]. To further verify the role of the *HSFA6b* gene in the UPR^mt^ signaling pathway and in the process of plant development, we performed an analysis of its overexpression lines and mutants’ developmental phenotypes. The wild type began bolting about 35 days after sowing, and compared to the wild type, the mutant plants exhibited early flowering by approximately 3 days. Overexpression of the *HSFA6b* transgene Arabidopsis did not exhibit significant differences (Figure 6a). Subsequently, we analyzed the senescence phenotype, where the wild type began to show senescence phenotypes after 6 weeks of sowing. To observe the significant differences, the phenotypes were recorded between 7 and 8 weeks after sowing; the results showed that compared to the wild type, the mutant plants exhibited an early senescence phenotype with a significant decrease in chlorophyll content in the leaves. In contrast, the overexpressing plants exhibited a delayed senescence phenotype, with the chlorophyll content in the leaves being significantly higher than that of the wild type (Figure 6b–d). In summary, *HSFA6b* mutation induces early flowering and premature senescence in Arabidopsis.

### 2.7. The UPR^mt^ Signaling Pathway Is Involved in Various Abiotic Stresses

**Figure 7:**
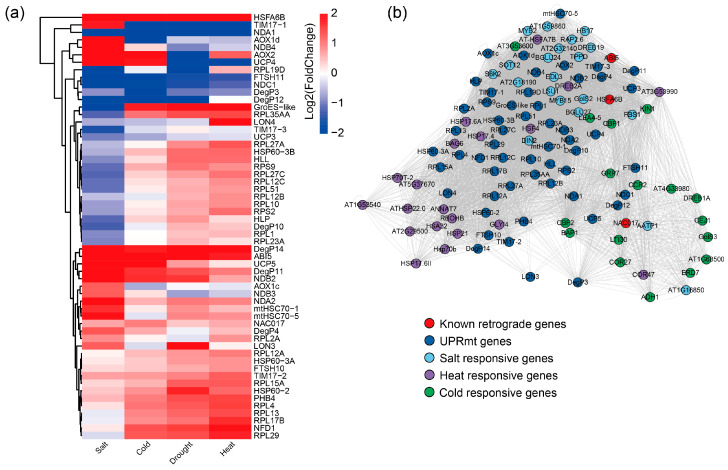
Expression pattern of UPR^mt^-associated genes under various abiotic stresses and network of UPR^mt^ gene and abiotic responsive genes: (**a**) Heatmap of UPR^mt^-associated genes under four abiotic stresses, including heat stress, salt stress, cold stress, and drought stress. The expression data were obtained from the GEO database with the IDs (GSE5620, GSE5621, GSE5624, and GSE5623). The colors indicate the log2 foldchange of those genes. The colors that are close to red indicate upregulation compared to the control, while the color close to blue indicates downregulation. (**b**) The correlation network between UPR^mt^-associated genes and abiotic stress-responsive genes. The network was constructed based on the Pearson correlation between the UPR^mt^-associated genes and abiotic stress-responsive genes. The gene pairs with the correlation coefficient > 0.7 were kept for the visualization using the software Cytoscape 3.10.0. The different colors of dots indicate the different types of genes.

We examined the expression of the UPR^mt^-associated genes under various abiotic stresses, including cold, drought, heat, and salt stress. The results showed that most UPR^mt^ genes were induced in response to these stresses (Figure 7a). Among these genes, *HSFA6b* was significantly upregulated across all stress conditions. Additionally, *ABI5*, a known key regulator of mitochondrial retrograde signaling, was also induced under these stresses. Moreover, the marker genes of UPR^mt^, such as *mtHSC70-1* and *mtHSC70-5*, were activated under all four stress conditions, particularly in response to salt stress. These findings suggest that UPR^mt^ may be involved in the response to abiotic stress. We also constructed a correlation network linking abiotic stress-responsive genes and UPR^mt^ genes based on the expression data. In this network, known mitochondrial retrograde regulators such as *ABI5* and *ANAC017* exhibited a high correlation with the abiotic stress-responsive genes. The gene *HSFA6b* was correlated with 56 genes, including 43 positively correlated genes and 13 negatively correlated genes (Figure 7b and Table S2). For instance, *HSFA6b* showed a strong positive correlation with *CBF1*, a key regulator under cold stress. These results indicate that UPR^mt^-associated genes may play a significant role in the response to various abiotic stresses.

## 3. Discussion

**Figure 8:**
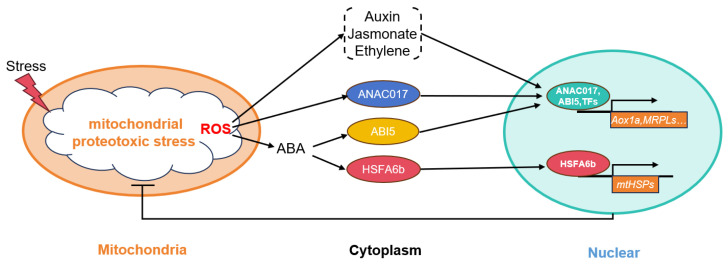
The model of heat shock factor *HSFA6b* mediating the UPR^mt^.

During evolution, plants, due to their sessile nature, gradually developed homeostasis mechanisms to adapt to environmental changes [26]. Mitochondria (known as the powerhouses of the cell) UPR^mt^ is a mitochondria-specific protein homeostasis mechanism that is essential for maintaining mitochondrial function. Activation of the UPR^mt^ pathway promotes the restoration of mitochondrial protein balance, protecting mitochondria from further damage and ensuring normal plant growth and development. In Arabidopsis, mutations in the mitochondrial ribosomal protein L1 (*MRPL1*) or treatment with doxycycline (a mitochondrial ribosome translation inhibitor) induce mitochondrial protein toxicity stress, leading to a transient oxidative burst in the cells. This activation triggers the hormone signals, such as ethylene, Jasmonate, and auxin. Once integrated into the nucleus, these various retrograde signals generate positive signals that promote the restoration of mitochondrial protein homeostasis, enhancing the expression of the genes involved in maintaining mitochondrial protein homeostasis, such as *MRPs* and *mtHSPs* [5]. Recent studies indicate that the mitochondrial retrograde signaling transcription factor *ANAC017* is involved in the transduction of UPR^mt^ signals. This transcription factor has been shown to localize to the endoplasmic reticulum (ER) and, under mitochondrial stress conditions, translocates to the nucleus, where it regulates the expression of mitochondrial stress-related genes such as *AOX1a*. Furthermore, gain- and loss-of-function mutants of *ANAC017* exhibit significant resistance or susceptibility, respectively, to mitochondrial stress-induced treatments, suggesting that ANAC017 plays a critical role in mitochondrial protein toxicity stress [17]. Additionally, UPR^mt^ signaling activates the ABA pathway, promoting the expression of UPR^mt^ genes, with *AOX1a* as a representative. *ABI5* (ABA Insensitive 5) is a key regulatory factor in ABA signaling that directly binds to the promoter of *AOX1a*, thereby enhancing its transcription [18]. In this study, we found that the ABA-sensitive heat shock transcription factor *HSFA6b* is involved in the transduction of UPR^mt^ signals. Following UPR^mt^ signal activation, HSFA6b is transported from the cytoplasm to the nucleus, where it binds to the promoter region of *mtHSPs* to enhance their expression. Our research further enriches the understanding of the UPR^mt^ signaling mechanism in plants, elucidating the activation of *mtHSPs* genes under mitochondrial protein toxicity stress and providing new insights into the interaction between hormonal signaling and UPR^mt^ signaling (Figure 8).

The *HSF* family is a crucial group of transcription factors in both animals and plants, playing a vital role in regulating cellular homeostasis [27]. *HSF* family genes are important in various stress conditions [28]. Compared to plants, the number of *HSFs* is lower in animals. Vertebrates have four *HSFs*, while fruit flies and Caenorhabditis elegans each contain one *HSF* [29]. In animals, HSF1 translocates to the nucleus during the UPR^mt^ process and activates the transcription of UPR^mt^ genes, serving as an important regulator of the UPR^mt^ signaling pathway [30]; recent research has indicated that the homologous gene of *HSFA6b* in maize is involved in the unfolded protein response triggered by heat stress [31]. *HSFA6b* in Arabidopsis plays a key role in response to ABA and heat tolerance; however, the study of *HSF* and its connection to UPR^mt^ in plants has yet to be explored. In this study, we combined reverse genetics to screen for the transcription factors that might participate in the regulation of the UPR^mt^ signal. Through promoter analysis of the *mtHSC70-5* gene and tobacco transcription activation assays, we identified the *HSFA6b* gene as an upstream transcriptional regulator of *mtHSC70-5*; *HSFA6b* mediates the UPR^mt^ signal, regulating mitochondrial protein homeostasis. Subcellular localization experiments demonstrated that HSFA6b responds to mitochondrial proteotoxic stress and exhibits nucleocytoplasmic shuttling. HSFA6b binds to the *mtHSPs* promoter and activates *mtHSPs* expression. Mutation of the *HSFA6b* gene disrupts UPR^mt^ signaling and promotes senescence in the *HSFA6b* Arabidopsis mutants.

In this investigation, we found that *HSFA6b* mediates the transduction of the mitochondrial UPR^mt^ signaling pathway. However, how HSFA6b senses mitochondrial stress and transmits the signal to the nucleus to activate downstream gene expression remains unknown. In earlier studies, after ABA treatment, the expression level of *HSFA6b* significantly increased, and HSFA6b partially but not completely translocated to the nucleus [21]. Therefore, we treated *HSFA6b:GFP* Arabidopsis seedlings with a high concentration of doxycycline—a mitochondrial ribosome translation inhibitor—and observed that the activation of the UPR^mt^ signaling pathway triggered the translocation of HSFA6b from the cytoplasm to the nucleus; this further confirmed that HSFA6b is an important signaling molecule in the UPR^mt^ pathway. Concurrently, after doxycycline treatment, the expression level of *HSFA6b* significantly increased (Figure 2a), indicating that *HSFA6b* responds to mitochondrial proteotoxic stress at both the transcriptional and protein levels. Previous studies have confirmed that mitochondrial proteotoxic stress induces a transient oxidative burst, activating the ABA signaling pathway. The downstream transcription factor *ABI5* in the ABA pathway directly binds to the promoter of *AOX1a* and enhances *AOX1a* promoter activity [18]. However, it has not been demonstrated how the mitochondrial heat shock proteins *mtHSC70-1* and *mtHSC70-5* are activated for expression in the UPR^mt^ pathway. In this study, we discovered and confirmed through reverse genetics that HSFA6b directly binds to the promoter regions of *mtHSPs* (*mtHSC70-1*, *mtHSC70-5*, and *HSP23.5*) to activate their expression. Combined with previous reports that *HSFA6b* functions as a downstream transcriptional regulator in the ABA signaling pathway [21,32], we propose the following hypothesis: mitochondrial proteotoxic stress induces a burst of reactive oxygen species, which activates the ABA signaling pathway, further enhancing the function of *HSFA6b* and leading to the activation of downstream *mtHSPs* gene expression. Of course, this hypothesis requires further experimental validation.

*MRPs* (mitochondrial ribosomal proteins) are a key determinant of lifespan in *C. elegans*. Reduced *MRP* expression and imbalanced mitochondrial protein translation lead to the activation of mtPQC and induce the UPR^mt^. *MRPs* have been shown to be important regulators of health and lifespan [33]. In our previous research, we demonstrated that mutations in the *MRPL1* (mitochondrial ribosomal protein L1) gene and doxycycline treatment disrupt the protein quality control system in Arabidopsis, activating the UPR^mt^ and extending the plant’s lifespan, while the root length decreases in the *mpl1* mutant or after Dox treatment. In this study, we found that after Dox treatment, the activation of the UPR^mt^ signal was partially suppressed in *hsfa6b* mutants, which led to an increase in root length under mitochondrial proteotoxic stress. Further investigation revealed that *HSFA6b* mutation shortens the lifespan, leading to early flowering and premature senescence, while overexpression of *HSFA6b* results in a delayed senescence phenotype. Our research enhances the understanding of how the UPR^mt^ signaling pathway regulates plant growth and development, providing new theoretical insights into the regulation of plant lifespan.

In conclusion, our results reveal a new signaling molecule in the UPR^mt^ pathway and preliminarily reveal the molecular mechanism by which *HSFA6b* mediates the plant UPR^mt^ signaling pathway. This provides a theoretical basis for further research on how the plant mitochondrial UPR^mt^ pathway regulates plant growth, development, and stress resistance.

## 4. Materials and Methods

### 4.1. Plant Materials and Growth Conditions

Arabidopsis Col-0 was used as the wild type, and all mutants and transgenic lines were in the Col-0 background. The *hsfa6b-1* (SALK_051357.2) mutant was ordered from AraShare, and *hsfa6b-3* (GK-513A02) was kindly provided by Dr. Xiaohong Zhu (Henan University, Kaifeng, China). The T-DNA mutants were identified using the three-primer method. For *hsfa6b-1*, two pairs of primers, *LBb1.3* and *RP*, as well as *LP* and *RP*, were used for genotyping (Figure S3a). For hsfa6b-3, two pairs of primers, LP and *8474*, as well as *LP* and *RP*, were used for genotyping (Figure S3c).

The *hsfa6b-2* is a CRISPR/Cas9 mutant, and its construction followed the method developed by Professor Qijun Chen [34]. The specific construction process is as follows: the two target sites on the *HSFA6b*, selected with the CRISPR-GE online program (http://skl.scau.edu.cn/, accessed on 8 November 2020), were separated by 150 bp. The sgRNA was synthesized and inserted into the pHEE401 vector, which was introduced into *Agrobacterium tumefaciens* EHA105 cells for Arabidopsis transformation. Transgenic Arabidopsis DNA was subsequently extracted, followed by PCR analysis and sequencing verification (Figure S3b). Homozygous mutants free of foreign DNA were isolated for use in further experiments.

For the creation of transgenic materials with *HSFA6b* overexpression, the *Xba1* and *Asc1* restriction sites on the pFGC5941-GFP vector were selected, and the *HSFA6b* CDS fragment was cloned into the expression vector pFGC5941-GFP using homologous recombination. The constructed plasmid was then introduced into the *Agrobacterium tumefaciens* strain EHA105, followed by transformation into Arabidopsis Col-0 using the floral dip method. Phosphinothricin was used as a selection marker to screen for positive homozygous transgenic plants, which were used for subsequent experiments. The primers used are listed in Supplementary Table S1.

For cultivation of Arabidopsis, place 100–150 Arabidopsis Col-0 wild-type seeds into each centrifuge tube. Add 1 mL of 70% ethanol and shake at 28 °C for 5 min. Remove the ethanol using a pipette, then add 0.5 mL of absolute ethanol and invert the tube 10 times. Quickly remove the absolute ethanol and air-dry the seeds in a laminar flow hood. Sow the dried seeds on ½ MS plates (2.17 g/L MS basal salt mixture, 1% sucrose, pH 5.8, 0.7% agar) and stratify them in the dark at 4 °C for 72 h. After stratification, transfer the plates to a growth room with a 16 h light/8 h dark cycle. After 9 days, transplant the seedlings into the soil for continued growth.

### 4.2. Stress Treatments

In this study, doxycycline treatment was used to activate the mitochondrial unfolded protein response in Arabidopsis. The long-term Dox treatment was as follows: Seven-day-old Arabidopsis seedlings were transplanted onto the ½ MS medium containing 10 μg/mL of Dox. Phenotypic analysis was performed two weeks later. For the short-term Dox treatment, seven-day-old Arabidopsis seedlings were transplanted onto fresh ½ MS solid medium containing 25 μg/mL of Dox, with ½ MS liquid medium without Dox as the control. The samples were collected at 3 h and 12 h post-treatment, total RNA was extracted, and qRT-PCR was conducted to detect the expression of UPR^mt^-related genes. The primers used are listed in Supplementary Table S1.

### 4.3. Transient Transcriptional Activation Assay in Tobacco

The promoter of the *HSP* genes was cloned into the vector pH2GW7-*LUC* and introduced into the *Agrobacterium tumefaciens* strain EHA105. The coding sequences (CDS) of selected transcription factors (*HSFA6b-AT3G22830*, *WRKY33-AT2G38470*, *ABI5-AT2G36270*, *GBF5-AT2G18160*, *bZIP16-AT2G35530*, *AREB3-AT3G56850*, *HY5-AT5G11260*, and *RAV1-AT1G13260*) were cloned into the expression vector pFGC5941 and introduced into the *Agrobacterium tumefaciens* strain EHA105. Equal volumes of Agrobacterium cultures containing pH2GW7-*HSPpromoter:LUC* and pFGC5941-*TF* (transcription factors) were mixed and infiltrated into 3-week-old tobacco plants grown under long-day conditions (16 h light/8 h dark). As a control, Agrobacterium containing pH2GW7-*HSPpromoter:LUC* and pFGC5941-*GFP* was co-infiltrated. After 48 h, the luciferase activity was measured using the IVScope 7000 plant in vivo imaging system, and the relative fluorescence intensity was calculated using the system’s software Clinx IVScopeEQ Capture (Shanghai Qinxiang Scientific Instrument Co., Ltd., Shanghai, China).

### 4.4. Subcellular Localization Experiment

The *HSFA6b* gene was fused with the green fluorescent protein (GFP) gene to generate the pFGC5941-*CaMV35S:HSFA6b:GFP* construct. This plasmid was introduced into the *Agrobacterium tumefaciens* strain EHA105 and transferred into Arabidopsis using the floral dip method. Positive transgenic plants were selected for the subcellular localization experiments. Five-day-old Arabidopsis seedlings, grown vertically on the medium in Petri dishes under long-day conditions (16 h light/8 h dark), were used for these experiments. Root cells were observed using a Leica SP8 confocal laser scanning microscope with 488 nm excitation.

### 4.5. RNA Extraction and qRT-PCR Analysis

Total RNA was extracted from whole seedlings using TRIzol reagent (VazymeR411-01, Nanjing, China). Then, refer to the reverse transcription kit (VazymeR333-01, Nanjing, China) for the method of reverse transcription cDNA synthesis. Fluorescent quantitative PCR was performed using the TB Green™ Fast qPCR Mix kit (Takara, Osaka, Japan). The amplification conditions were as follows: predenaturation 95 °C 30 s, PCR reaction 95 °C 5 s, 60 °C 30 s, and 40 cycles. The results were calculated by the 2^−ΔΔCt^ method, and the primers used are listed in Supplementary Table S1.

### 4.6. Chlorophyll Measurement

Select plants were grown in the same greenhouse with consistent growth periods as follows: cut the leaves of the same age, remove the main leaf vein, and slice the leaves into strips less than 2 mm wide. Weigh 0.5 g of the leaf strips and place them into a test tube, then add a mixture of 20% ethanol and 80% acetone to reach a final volume of 10 mL. Seal the tube and incubate it in the dark until the leaves are completely decolorized. Afterward, extract the supernatant and measure the absorbance at 663 nm and 645 nm using a UV spectrophotometer. Calculate the concentrations of chlorophyll a and b using the formulas: Ca = 12.72 × A663 − 2.59 × A645 and Cb = 22.88 × A645 − 4.67 × A663, where A663 and A645 represent the absorbance values at 663 nm and 645 nm, respectively. Ca and Cb denote the concentrations of chlorophyll a and b in mg/L. The total chlorophyll concentration is then calculated as Ct = Ca + Cb = 8.05 × A663 + 20.29 × A645. Finally, determine the chlorophyll content of the sample (mg/g fresh weight) using the formula Ct × V/W, where V is the volume of the extract, and W is the mass of the leaf tissue.

### 4.7. Yeast One-Hybrid Assay

The yeast one-hybrid assay was conducted following the protocol described by Lin et al. [35], with the detailed steps as follows: The full-length coding sequence of *HSFA6b* was amplified and cloned into the pB42AD vector at the EcoRI restriction site using homologous recombination, generating the pB42AD-*HSFA6b* construct. The promoter region of the *mtHSP* gene containing the HSE element was cloned into the pLacZ vector to create the reporter plasmid. The pB42AD and pLacZ empty vectors were used as negative controls. Various combinations of effector and reporter plasmids were transformed into the yeast strain EGY48 using the LiAc method. Transformants were cultured on a double-dropout (SD/-Trp/-Ura) medium, and positive clones were confirmed by PCR. These clones were then transferred to a selective medium containing X-Gal (SD/-Trp/-Ura+X-Gal) for blue color development. Primers used in the yeast one-hybrid assay are listed in Supplementary Table S1.

### 4.8. ChIP-qPCR Analysis

The ChIP experiment was conducted as described by He et al. [36], *HSFA6b:GFP* transgenic *Arabidopsis* seeds were sown on ½ MS plates (2.17 g/L MS basal salt mixture, 1% sucrose, pH 5.8, 0.7% agar), and 12-day-old seedlings were treated with 25 μM Dox in the ½ MS liquid medium for 3 h. For each sample, 1.2 g of seedlings were harvested and cross-linked under vacuum in 1% (*v*/*v*) formaldehyde solution for 15 min; chromatin was then purified and sonicated, followed by centrifugation to collect the fragmented chromatin. The purified chromatin was incubated overnight at 4 °C with 10 μg of anti-GFP antibody (Abcam, Shanghai, China, ab290) and 50 μL of protein A Agarose (Abcam, ab193255). The samples without antibodies were used as negative controls. The chromatin samples were washed sequentially with low-salt buffer (50 mM Tris-HCl, pH 7.4, 150 mM NaCl, 2 mM EDTA, 0.5% Triton X-100), high-salt buffer (50 mM Tris-HCl, pH 7.4, 500 mM NaCl, 2 mM EDTA, 0.5% Triton X-100), and wash buffer (50 mM Tris-HCl, pH 7.4, 50 mM NaCl, 2 mM EDTA). After reverse cross-linking, the samples were digested with proteinase K, and DNA was purified using phenol: chloroform: isoamyl alcohol (25:24:1, *v*/*v*/*v*) for the ChIP-qPCR analysis. The relative enrichment was calculated as IP/Input. The experiments were conducted in three biological replicates, and the primers used for ChIP-qPCR are listed in Supplementary Table S1.

### 4.9. Transcriptome Analysis and Correlation Network Construction

The gene expression data of Arabidopsis under different abiotic stresses were obtained from the GEO database (https://www.ncbi.nlm.nih.gov/geo/, accessed on 27 September 2024). The control plants were downloaded with ID (GSE5620). The abiotic stresses included cold stress (GSE5621), drought stress (GSE5624), and salt stress (GSE5623). The list of abiotic responsive genes was identified as the top 20 differentially expressed genes under the corresponding stress. The correlation between the UPR^mt^ genes and abiotic stress-responsive genes was conducted with a Pearson’s correlation > 0.7. The network was visualized with Cytoscape software.

## Data Availability

Data are contained within the article.

## Supplementary Materials

The following supporting information can be downloaded at: https://www.mdpi.com/article/10.3390/plants13223116/s1, Figure S1:Reporter gene screening for mitochondrial UPR^mt^ signaling pathway; Figure S2: Binding sites of candidate transcription factors regulating the expression of *mtHSC70-5*; Figure S3: Identification of *hsfa6b* mutants; Table S1: Primers and Gene ID; Table S2: The correlation coefficient between UPR^mt^ -aassociated genes and abiotic stress- responsive genes.
