# Supplementary material for: Heat Shock Factor HSFA6b Mediates Mitochondrial Unfolded Protein Response in Arabidopsis thaliana
Source: Plants (Basel). 2024 Nov 5;13(22):3116. doi: 10.3390/plants13223116 (PMC11597222; doi:10.3390/plants13223116)
Supplement: Supplementary file 1 [file plants-13-03116-s001.zip › Supplemental_Figures.pdf]

Supplemental Figures

Supplemental Figure S1.

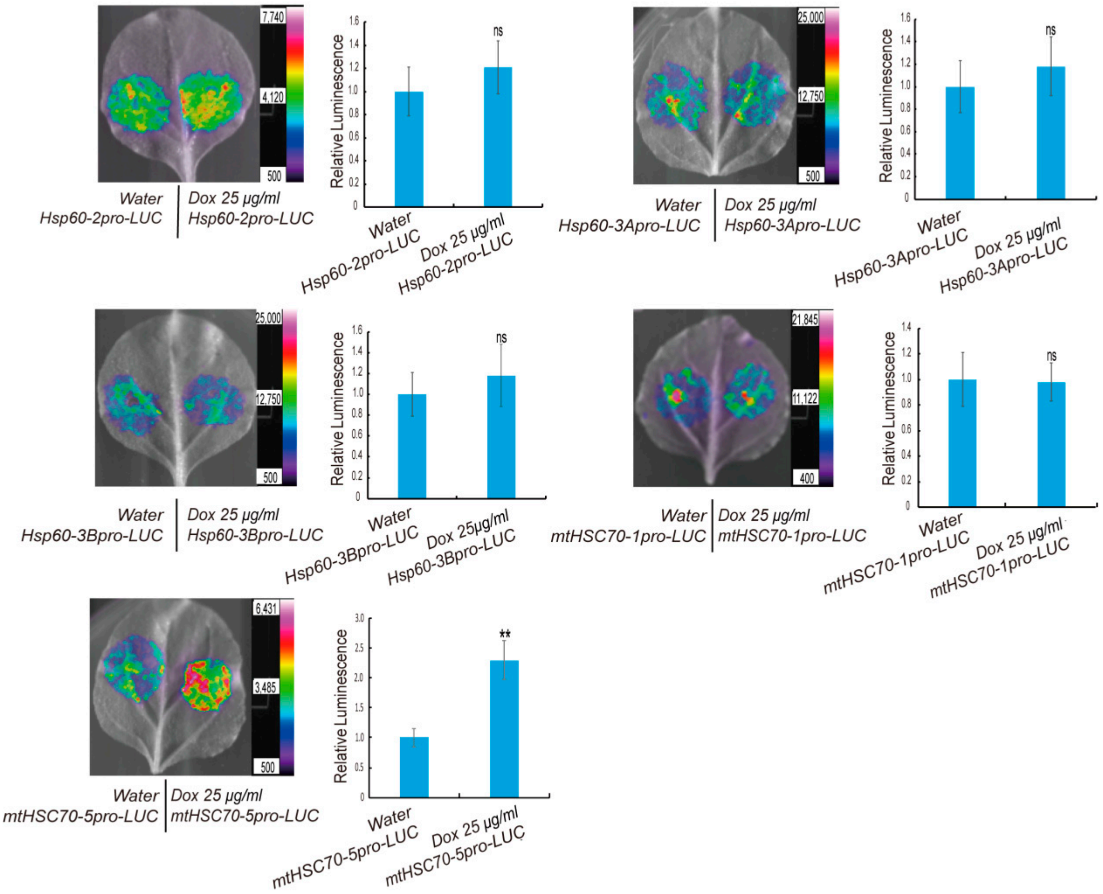

Supplemental Figure S1. Reporter gene screening for mitochondrial UPR^{mt} signaling pathway : In each transcription activation experiment, 10 tobacco leaves were injected. The images in the figure represent the experimental setup, with the mean relative fluorescence values plotted for the experimental and control groups. The promoter cloning lengths for the target genes in the genome are as follows: *Hsp60-2 promoter* (405 bp), *Hsp60-3A promoter* (628 bp), *Hsp60-3B promoter* (1260 bp), *mtHSC70-1 promoter* (438 bp), and *mtHSC70-5 promoter* (944 bp). The experiment was conducted with three biological replicates, and paired two-sample t-tests on the average values were used to analyze significant differences (ns indicates no significant difference with $P > 0.05$, ** represents $0.001 < P < 0.01$). Error values are presented as standard errors.

Supplemental Figure S2.

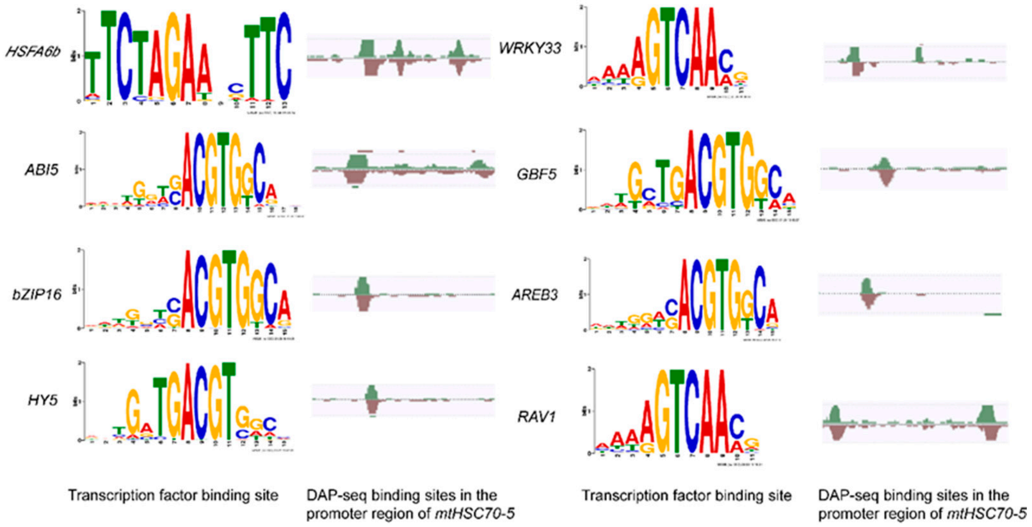

Supplemental Figure S2. Binding sites of candidate transcription factors regulating the expression of *mtHSC70-5* :Transcription factors with DAP-seq binding sites within 1 kb of the *mtHSC70-5 promoter* region are considered potential regulators of *mtHSC70-5*.

Supplemental Figure S3.

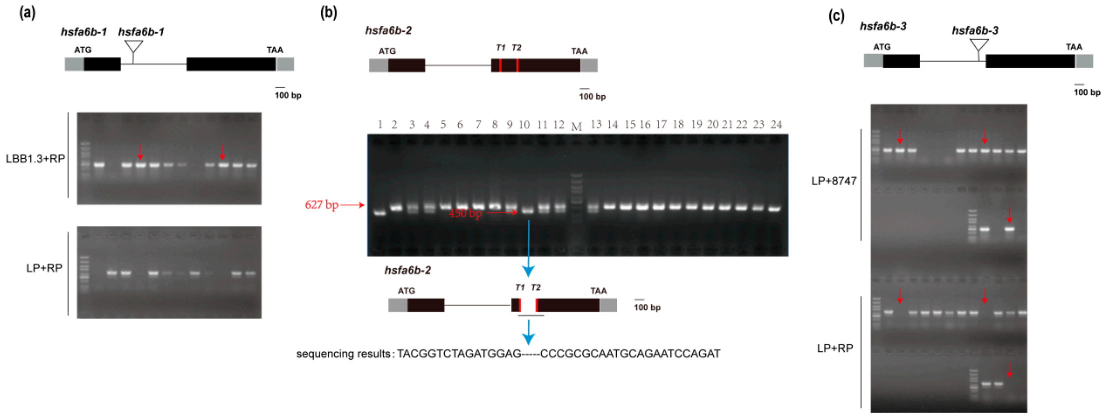

Supplemental Figure S3. Identification of *HSFA6b* mutants: **(a)** Diagram of the insertion site in the *hsfa6b-1* mutant and PCR identification results; **(b)** Diagram of the insertion site in the *hsfa6b-2* mutant and identification results: *hsfa6b-2* is a crisper/Cas9 mutant, in the schematic diagram, T1 and T2 represent two designed CRISPR target sites, which are spaced 150 bp apart on the same exon. When both sites are activated simultaneously, they will result in a gene deletion of approximately 150 bp, leading to a gene mutation. This will be further identified through PCR and sequencing methods, the figure shows the PCR identification results and sequencing results; **(c)** Diagram of the insertion site in the *hsfa6b-3* mutant and PCR identification results; The locations of T-DNA (triangles), UTR (gray box), intron(straight line) and exon (black box) are shown.
